# Equity in use of maternal health services in Western Rural China: a survey from Shaanxi province

**DOI:** 10.1186/1472-6963-14-155

**Published:** 2014-04-05

**Authors:** Yuan Shen, Hong Yan, Klemetti Reija, Qiang Li, Shengbin Xiao, Jianmin Gao, Zhongliang Zhou

**Affiliations:** 1 Department of Epidemiology and Health Statistics, School of Public Health, Xi’an Jiaotong University College of Medicine, Xi’an, Shaanxi 710061, P.R. China; 2 THL, National Institute for Health and Welfare, P.O. Box 30, Helsinki FI-00271, Finland; 3 School of Public Policy and Administration, Xi’an Jiaotong University, Xi’an, Shaanxi 710061, P.R. China

**Keywords:** Equity, Maternal and child health service, Economic situation

## Abstract

**Background:**

The 20^th^ century was marked by a significant improvement in worldwide human health and access to healthcare. However, these improvements were not completely or uniformly distributed among, or even within, nations. This study was designed to assess the use of maternal health services by pregnant women in China, with a focus on the inequity related to family income level.

**Methods:**

Two population-based cross-sectional surveys were carried out in the Zhenan and Lantian counties in March 2007 and from December 2008 to March 2009. A total of 2562 women completed the questionnaires, including 948 who were pregnant in 2006 and 1614 from 2008–2009. The concentration index (CI) was calculated and used to analyze the parameters of maternal health care in the two counties surveyed.

**Results:**

The responses in both 2006 and 2008–2009 indicated a bias towards higher (rich) economic statuses for the use of maternal and child health services. The CI of ‘delivery at health facility’ was 0.0206 (95% confidence interval between 0.0114 and 0.0299) for 2006 and 0.0053 (95% confidence interval between 0.0015 and 0.0091) for 2008, which represented a statistically significant inequity for women of lower (poor) economic statuses. Similar CI was observed in ‘receiving antenatal care within 12 weeks’ for 2006 (CI_2006_ = 0.0956, 95% confidence interval between 0.0516 and 0.1396). The CIs of ‘postnatal visit’ and ‘postnatal visit >3-times’ was positive (except for 2006), indicating that the poor used postnatal care less than the non-poor. In 2008, poor women had C-sections more often than non-poor women (CI_2008_ = −0.0629, 95% confidence interval between-0.1165 and −0.0093), but such a difference was not observed in 2006.

**Conclusions:**

In 2006 and 2008, the use of maternal health services in western rural China was significantly unequal between pregnant women of poor and non-poor economic statuses. Financial support that enables poorer pregnant women to use health services will be beneficial. Utilization of maternal healthcare services can be improved if out-of-pocket expenses can be minimized.

## Background

The 20^th^ century was characterized by a significant improvement in public health, both in developing and developed nations. According to the *World Health Report* published by the World Health Organization (WHO) in 2000, China had a serious inequity in offering health resources to its citizens [1]. Stratified analysis of the situation in Morocco, another currently developing country, identified the area-based differences in access to basic public welfare services, such as education and health care [2].

In the year 2000, the WHO and United Nations developed a set of Millenium Development Goals to combat the most serious threats to human life and welfare; maternal health was ranked among one of the most important issues to address and significantly improve by 2015 [3]. The issue of maternal health has been extensively studied throughout modern history and is known to be correlated with many social, cultural and economic factors [4,5]. Undoubtedly, poverty is a determining factor of rates of complications in pregnancy and poor pregnancy outcomes [6]. In China, the marked imbalance of economic development has affected access to and use of maternal and child health (MCH) services, generating a severe inequity among various economic statuses. Chinese national health statistics from 2000 revealed the existence of an inequity in maternal mortality between different provinces and between central and western regions [7]. Furthermore, this statistically significant inequity remained up to 2005 [7]. Inequity of maternal mortality also remained between the central and western regions. These studies largely relied on concentration curves to determine where inequity (a data point above the line of equality) was greatest or lowest, and found that western China led the trend, followed by middle and eastern China [8].

Several Chinese studies have shown that standard health services during pregnancy can reduce the rate of maternal mortality [9,10]. National monitoring of maternal health care from 1989 to 1995 showed that lower economic levels were associated with higher maternal mortality rates [9]. Families with per capita household incomes of less than 20 renminbi (RMB) per day (1 RMB = 3.08 United States dollar (USD)) had a maternal mortality rate of 0.2171/10,0000 persons, which was 6.1 times that of families with per capita household incomes of more than 50 RMB (7.69 USD) [9]. Improving the quality of services is the one of the fundamental objectives of maternal health services and will contribute to reducing maternal mortality. National data of maternal mortality from 1996 to 2000 showed that, in cases of maternal death, 28.9% of pregnant women did not receive antenatal care and that the majority of those cases were from rural areas. Specifically, 28.4% of urban-dwelling pregnant women received up to eight antenatal visits, while 46.1% of rural pregnant women received up to five visits [10]. Analysis of the secondary data sources has suggested that, despite overall improvements in the population’s health status, the economic and health system policy reforms are actually promoting increased inequities in health care; it was shown that the lowest income quintiles in both urban and rural areas received less health care in 1998 than in 1993 [11].

In recent years, the situation in urban areas nationwide has improved slightly, but in certain regions, such as in northern China, the inequity continues to deteriorate. The concentration index (CI) rose from 0.015 in 1994 to 0.295 in 2000 in urban areas, indicating increased inequity. Over the same period, the CI for rural areas rose from 0.002 in 1994 to 0.026 in 2000, which also indicates increasing inequity in rural areas [11]. A large-scale investigation of 1000 counties indicated that both the maternal mortality rate (MMR) and the hospital delivery rate were affected by economic level of individuals; specifically, MMR was found to be inversely proportional to income per capita and hospital delivery rate [11]. Similar results were reported by two other studies of rural areas in China [12,13].

In 2003, the Chinese government implemented a nationwide pilot project (titled as the “New Cooperative Medical Scheme” (NCMS) that aimed to reduce illnesses and deaths related to poverty by improving rural residents’ access to health care services [14,15]. Starting in 2007, the cost of delivering at a healthcare facility in China has been covered by the NCMS, and this aspect of the program was developed to further lessen the inequities that disfavor the poor. From 2007 to 2010, a project known as the “Structural Hinders to and Promoters of Good Maternal Care in Rural China” (CHIMACA) was developed to address the quality of health providers and their services, and was introduced to the Zhenan and Lantian counties in the Shaan’xi province. One of the key objectives of CHIMACA was to evaluate the equity in utilization of MCH services among pregnant women from various socio-economic backgrounds.

The present study was designed to present the inequity in use of MCH in relation to the health system reform. Quintile ratios and concentration curves and indices were calculated to gain insight into maternal and child health in the Shaanxi province during 2006 to 2009.

## Methods

The study involved two counties, Zhenan and Lantian, in the Shaanxi province in northwest China. Both counties are representative of poor, rural regions, in terms of national per capita net income rankings. The average per capita income is less than 2,700 RMB (397.62 USD) [16], and the major industry and work type is farming. Two population-based cross-sectional surveys were carried out in Zhenan and Lantian County in March 2007 and in December 2008 to March 2009 as a part of the CHIMACA project (to assess structural barriers and promoters of good maternal care in rural China). One-third of the villages in the townships were selected randomly by using the Probability Proportionate to Size (PPS) sampling method. For the 2006 data, the target survey population was all women who were residents in the selected villages and who had given birth between January 1 and December 31 in either county. For the 2008–2009 data, the target survey population was all women who were residents in the selected villages and who had given birth between April 2008 and March 2009.

We attempted to collect contact information (i.e. telephone number and home address) for each of the women who had delivered according to our date criteria. Initial contact was made posting letters to the familial residence address, which requested an in-person/in-home consultation with the woman and provided detailed information about the objective. If the woman was not at home at the scheduled interview time, the research team either chose to wait until she returned (if she was not far away) or rescheduled the appointment. If the woman was unamenable to scheduling the interview, she was excluded from the study.

A total of 2562 women completed the questionnaires, including 948 in 2006 and 1614 in 2008–2009. In 2006, the PPS target population list was not obtained, but the interviewers estimated this value by considering the reported number of newborns in 2005 that were born to women who fit the study criteria but were not reached by initial recruitment efforts (this proportion of women was around 35%); since the target number of newborns in 2005 was about 1800 and migrant women made up about 20% of the women’s population, the target population was estimated to be around 1440. In 2008, the response rate was 76%. The most common reasons for non-response in 2008 were: no one at home at the time of survey (10.8%), working outside of their hometown (11.4%), transportation or other problems (1.8%), no or incomplete contact information (0.3%), or refusal to be interviewed (0.1%).

For both surveys, Ph.D. students and medical students were recruited to conduct in-person interviews and to collect data. The surveyors were organized into two groups for on-site surveying in either Lantian or Zhenan County. Each group was composed of 4–6 members from Xi’an Jiaotong University and 2–3 members from the respective county’s health care facilities. Prior to the survey, village doctors and MCH staff recruited women who fit the study criteria by telephone or home visit. Interviewers had undergone training by the Shaanxi research team of the CHIMACA project, which had included practical interview sessions and theoretical lectures. The questionnaire that was administered included the women’s demographic and socioeconomic background, history of pregnancy, utilisation of and expenditures on maternal health care for the latest pregnancy.

Ethical approval for the CHIMACA project was obtained from the International Centre for Reproductive Health (ICRH) at Ghent University, Belgium, and a local approval was obtained from the ethics committee of Xi’an Jiaotong University. Invitation letters were given to all study participants prior to the survey being conducted and included assurances that participation was voluntary. The women who agreed to participate in the study were interviewed at home or in a public setting; all interviews were carried out by trained researchers and Ph.D./medical students using a structured questionnaire. All participants provided written, informed consent prior to study participation.

The disparities in China’s social and economic development, along with the unequal access to and use of health services promote the observed imbalance of health problems among the different socio-economic levels in society. The outcome measures had been selected before we read the participants’ answers on the questionnaires, by using the following criteria: clinically important, successful question formulation, sufficient information, and appearance in both of the two surveys.

The utilization rate of antenatal care, number of postnatal visits, and delivery place were used as indicators of health service use. Maternal outcomes (i.e. gravidity history and delivery outcome) were used to indicate the equity of health outcome. The proportion of delivery at a health facility was calculated as the number of women giving birth in health facilities (including township, county and higher-level hospitals) divided by all delivered women in the survey. The proportion of delivery at a county- or higher-level health facility was calculated as the number of women who gave birth in a county- and province-level health facility divided by all delivered women in the survey. The proportion of home delivery was calculated as the number of women who gave birth at home divided by all delivered women in the survey. The proportion of antenatal care (or ≥5 times) was calculated as the number of women who received antenatal care (or ≥5 times) divided by all pregnant women in the survey. The proportion of antenatal care within 12 weeks of pregnancy was calculated as the number of women who received their first antenatal care within 12 weeks divided by all pregnant women in the survey. The proportion of postnatal care (or ≥3 times) was calculated as the number of women who received postnatal care (or ≥3 times) divided by all delivered women in the survey. The proportion of C-section was calculated as the number of women who underwent a C-section delivery divided by all delivered women. The family income was calculated by summing the income (in RMB) for every family member per year, with the data classified into five equal groups for each year.

Several methods are available to evaluate equity in healthcare access, including CI, the Gini coefficient (which focuses on funding of health facilities), the Le Grand method (which focuses on the monetary fees associated with use of a health service), and logistic regression. Nonetheless, CI has been used widely in studies of healthcare inequity conducted by the World Bank (Development Report), the WHO (World Health Report), and China’s Health Service (National Survey Report). The CI was defined with reference to the concentration curve, on which the x-axis referred to the cumulative percentage of the sample, ranked by living standards beginning with the poorest and on which the y-axis to the cumulative percentage of the health variable corresponded to each cumulative percentage of the distribution of the living standard variable. The CI was defined as twice the area between the concentration curve and the line of equality (the 45° line running from the bottom-left corner to the top-right corner). In the case of no income-related inequality, the CI would be zero. The convention is that the index will have a negative value when the curve lies above the line of equality, indicating disproportionate concentration of the health variable among the poor, and a positive value when it lies below the line of equality. Therefore, if the health variable was ‘bad’, such as ill health, a negative value of the CI was expected to indicate that ill health was higher among the poorer people [17]. The CI was computed by the standard method of the “convenient covariance” formula: $C=\frac{2}{\mu }\mathrm{cov}\left(h,r\right).$ The CI that is computed from the concentration curve assumes values between −1 and +1. In the absence of inequalities (the concentration curve coinciding with the diagonal), the value of the concentration index is then expected to be zero. In this study, the CI and concentration curve were used to analyze the equity of maternal health care in the surveyed counties, and the *t-*test statistic was used to test the significance of the CIs.

## Results

For both surveys, the average age of participants was 26 years old. The median number of years of education was seven in 2006 and nine in 2008–2009. Median income was 9,000 RMB (1384.62 USD) in 2006 and 12,000 RMB (1846.15 USD) in 2008–2009. The characteristics for each year are summarized in Table 1.

**Table 1:** Background characteristics of Chinese rural women in 2006 and 2008-2009* (n,%)

		**2006 (n = 948)**			**2008-2009 (n = 1614)**	
Age in years						
15–24		295	36.9		656	40.6
25–29		249	31.2		564	34.9
30–49		255	31.9		394	24.4
Education**						
Illiterate		41	4.6		33	2.2
Primary school		212	23.6		222	14.5
Secondary school or higher		645	71.8		1279	83.4
Annual family income*** in RMB	9000			12000		
Poorest	5000	255	26.9	8000	418	25.9
2^nd^ poorest	7000	129	13.6	10000	353	21.9
Middle	10000	239	25.2	15000	311	19.3
4^th^ richest	15000	186	19.6	20000	289	17.9
Richest	15000+	139	14.7	20000+	243	15.1
History of abortion or stillbirth						
No		802	84.0		558	69.5
Yes		153	16.0		245	30.5

*n(%).

**Illiterate refers to women who did not receive any education or received <1 year; primary school refers to women who received education for ≤6 years; secondary school or higher refers to women who received education for >6 years.

***Annual family income refers to the total income of the family last year.

Utilization rates of antenatal care, postnatal visits, and delivery place are commonly used as indicators of health service use. Each of these parameters was surveyed, and the results are presented in Table 2. The proportion of births that were delivered at a health care facility, as opposed to deliveries in the home, was 94.1% in 2006 and 97.5% in 2008–2009. The CI was 0.0206 in 2006 and 0.0053 in 2008–2009. This difference showed statistical significance, which indicated that there was inequity in delivery at a health care facility both in 2006 and 2008–2009. The proportion of deliveries that occurred at county- or higher-level hospitals was 25.8% (2006) and 73.7% (2008–2009), while that for home delivery was 5.0% (2006) and 1.5% (2008–2009). The CIs of these indicators showed statistical significance, which indicated that there was inequity both in 2006 and 2008–2009, with poorer women using hospital delivery less and having a higher rate of home delivery than their non-poor counterparts.

**Table 2:** Distribution and CI in use of maternal and child health services by Chinese women in 2006 and 2008-2009

**Health care service**	**Year**	**Income level,%**						**CI **^**a**^**(95% confidence interval)**
		**Poorest**	**2**^**nd**^	**Middle**	**4**^**th**^	**Richest**	**Total**	
Delivery at health facility	2006	88.6	91.9	96.6	97.2	97.2	94.1	0.0206(0.0114, 0.0299)*
	2008	94.3	98.3	98.0	98.0	99.7	97.5	0.0053(0.0015, 0.0091)*
Delivery at county- or higher-level health facility	2006	16.1	23.9	22.2	26.1	38.8	25.8	0.1372(0.0711, 0.2033)*
	2008	66.1	74.2	71.5	74.5	81.6	73.7	0.0309(0.0136, 0.0481)*
Home delivery	2006	11.1	7.2	3.3	2.8	2.2	5.0	−0.3566(−0.5417, −0.1715)*
	2008	3.7	1.0	1.3	1.3	0.0	1.5	−0.3756(−0.7216, −0.0295)*
Any antenatal care	2006	96.1	97.2	97.2	98.3	99.4	97.7	0.0074(0.0016, 0.0133)*
	2008	96.6	99.0	99.0	100.0	100.0	99.2	−0.0063(−0.0092, −0.0033)*
Antenatal care ≥5 times	2006	48.3	43.3	55.2	60.2	77.5	57.1	0.1305(−0.2791, 0.5401)
	2008	49.8	66.4	68.8	64.0	80.9	66.8	0.0785(0.0574,0 .0995)*
Antenatal care within 12 weeks of pregnancy	2006	38.7	32.4	45.9	48.0	53.9	43.0	0.0956(0.0516, 0.1396)*
	2008	68.4	75.3	74.1	77.2	83.9	75.8	0.0365(0.0197, 0.0532)*
Any postnatal visit	2006	50.6	33.9	58.3	33.3	43.6	42.5	0.07105(0.0282, 0.1139)*
	2008	45.6	51.7	43.6	47.6	52.2	48.4	0.0355(0.0046, 0.0664)*
Postnatal visit ≥3 times	2006	32.8	19.4	26.7	10.0	26.8	22.1	0.0602(−0.0091, 0.1294)
	2008	28.9	27.9	23.0	26.9	30.9	27.9	0.0527(0.0042, 0.1014)*
Antenatal care ≥5 times and postnatal visit ≥3 times	2006	22.2	8.9	17.8	6.7	23.5	15.2	0.1354(0.0461, 0.2248)*
	2008	16.8	21.5	19.1	19.8	26.1	21.0	0.1172(0.0583, 0.1761)*
C-section	2006	18.9	18.9	16.1	23.3	23.5	20.0	0.0426(−0.0329, 0.1182)
	2008	24.9	24.3	24.0	23.5	20.1	23.3	−0.0629(−0.1165, −0.0093)*

CI ^a^concentration index.

*P < 0.05, for t-test comparison with CI = 0. A negative value of the CI was expected to indicate a higher rate among the poorer people.

The proportion of women who had participated in any amount of antenatal care was over 95% during 2006 and 2008–2009 (Table 2). An inequity was found in the improvement of use of maternal health services, with rich women using significantly more antenatal care in 2006 (CI = 0.0074) and the opposite in 2008–2009 (CI = −0.0063). Having ≥5 antenatal visits was less likely among the poorest women in 2008–2009, and the CI of ≥5 antenatal visits was 0.1305 in 2006 and 0.0785 in 2008–2009; although the difference between poor and non-poor women was insignificant in 2006, it was significant in 2008. Similar was observed for antenatal care received within 12 weeks of birth. The proportion of postnatal care, as well as the proportion of ≥3 postnatal visits, was 42.5% and 22.1% in 2006 and 48.4% and 27.9% in 2008–2009. However, poor women were found to have used ≥3 postnatal care significantly less than non-poor women in 2008–2009 (CI = 0.0527).

The indicators of C-section (Table 2) of 2006 showed no significant difference with the line of equity, while that of 2008–2009 was above the equity line. The differential pattern signified that non-poor women had C-sections no more than poor women in 2006, but this appeared in 2008–2009.

CIs of gravidity history and outcome of delivery, including number of pregnancies and proportion of low birth weight showed no significant difference (Table 3). The distribution of full-term births indicated no inequity in the two time periods examined, but the number of children born to a mother showed inequity among the economic groups. The CI was −0.134 (95% confidence interval between −0.1796 and −0.0885) for 2006 and −0.0728 (95% confidence interval between −0.1075 and −0.0381) for 2008. Specifically, the poorer a woman was the more likely to have more babies.

**Table 3:** Distribution and CI of history of pregnancy and delivery outcomes among Chinese women in 2006 and 2008-2009

**Gravidity and outcomes**	**Year**	**Income level,%**					**Total**	**CI **^**a**^**(95% confidence interval)**
		**Poorest**	**2**^**nd**^	**Middle**	**4**^**th**^	**Richest**		
Number of pregnancies ≥3	2006	14.4	11.7	16.7	10.6	10.1	12.6	−0.0706(−0.1695, 0.0282)
	2008	19.4	25.7	15.3	17.3	20.7	19.4	−0.0075(−0.0915, 0.0764)
Number of children ≥2	2006	51.7	49.4	45.0	35	24.6	40.6	−0.1340(−0.1796, −0.0885)*
	2008	57.0	37.2	42.6	40.6	34.8	41.8	−0.0728(−0.1075, −0.0381)*
Birth weight <2500 g	2006	5.0	7.2	3.3	5.0	3.4	4.6	0.0975(−0.0737, 0.2687)
	2008	2.8	1.7	4.1	2.7	1.7	2.8	0.1554(−0.0324, 0.3433)
Gestational weeks ≥37	2006	85.6	86.1	88.9	83.3	87.2	86.4	−0.0013(−.0165, 0.0138)
	2008	94.5	91.7	97.2	95.8	95.4	94.6	0.0061(−0.0076, 0.0199)

CI ^a^concentration index.

*P < 0.05, for *t*-test comparison with CI = 0. A negative value of the CI was expected to indicate a higher rate among the poorer people.

## Discussion

Our findings indicate that in both 2006 and 2008–2009, there was inequity in deliveries occurring at a health care facility of county- or higher-level hospitals. However, the proportion of home delivery was not equal among low- and high-income families, with higher income families representing the larger portion of births that occur in health care facilities in both years. The obvious inequity between the poor group and the wealthy group reflects the fact that the costs associated with delivery at a healthcare facility, such as non-delivery related hospital fees and travel or time away from the home and work for other family members or caretakers, all of which remain a burden for low-income families.

In addition, the same condition was observed for use of attending ≥5 antenatal visits and partaking in postnatal care, as well as the proportion of ≥3 postnatal visits attended. The poor women remained less likely to attend ≥5 antenatal visits, to receive antenatal care within the recommended 12 weeks, or to carry out the recommended ≥3 postnatal visits, as compared to their non-poor counterparts. Differences in carrying out postnatal visits were also remarkable among the different economic groups. This may reflect an inhibition produced by out-of pocket expenses. In general, postnatal visits are not very common in China [18], and this was also observed in our study. Usually, poorer women live further away from hospitals, which represents an additional challenge in traveling to the facility and results in less postnatal visits. Moreover, the poorer population often has a less robust consciousness of self-care, due to the limited education compared to the wealthier population; therefore, they tend not to take the initiative to seek health care services. Similar results have been found in another study on basic health service utilization in China [19].

An inequity in the use of C-sections appeared in 2008–2009, which indicated that poor women were inclined to take C-section than their non-poor counterparts. This outcome was also supported by other research carried out in rural China by another team. In that study it was shown that from 1993 to 2010, 129,219 of the 49,6054 cesarean deliveries were carried out upon maternal request (CDMR). The prevalence rates of cesarean delivery and CDMR were reported as 37.6% and 10.0%, respectively; moreover, the proportions of CDMR for all cesarean deliveries were found to be significantly increased in both the north and south regions [20]. A subsequent study also indicated that the proportion of CDMR continues to rise [21].

Among all of the indicators examined in our study, the rate of home delivery showed the greatest inequity. In China, the home delivery rate is generally very low (5% in 2006 and 1.5% in 2008 from our study), and the main factors associated with home delivery have been reported to be the mother’s economic conditions, level of education, and location [22]. Pregnant women who are poor generally live farther away from health facilities than their wealthier counterparts, and have lower levels of education. Comparison to other indicators in our study showed that home delivery is the strongest correlated factor to a mother’s economic situation. Moreover, home delivery was mostly concentrated within the poorer population sample and showed the most robust inequity in our analysis.

Another intriguing result of our study was that CIs of gravidity history and outcome of delivery, including number of pregnancies, showed no significant, correlation with proportion of low birth weight and gestational weeks ≥37. This finding agreed with previous reports, in which most of the indicators were associated with mother’s age and inter-pregnancy intervals [23,24]. The mother’s economic situation has not been reported as an associated factor.

The improvement on utilization of maternal and child health we observed is in accordance with the Chinese government’s goals of increasing access to and utilization of healthcare by all citizens, regardless of economic status. It is also in line with trends observed in previous studies of western rural China, especially in undeveloped areas [25-27]. The Chinese national initiatives of NCMS and MCH were established with the principal aim of lessening inequities in human health care that disfavor the poor. NCMS was introduced in counties of the Shaanxi province in 2004, and was gradually expanded throughout the region. One feature of NCMS was to cover the costs of delivery at health care facilities, and this may have encouraged a large amount of women to eschew home delivery. In addition, the CHIMACA project has also helped to improve the quality of health service and health education in local areas, to a certain degree. In 2008, some local programs were introduced to cover the entire cost of hospital delivery at a health facility. This more comprehensive initiative further promoted healthcare utilization and effectively improved the rates of utilization of hospital delivery among poor women while reducing the inequity in maternal health care utilization in rural China [28].

In recent years, a substantial amount of national projects have been launched to ameliorate maternity services. These public health initiatives have improved the access to and utilization of health services in western rural China. In addition, it has also become evident that regular use of antenatal services can improve delivery outcomes, both for the mother and the child. As such, the Chinese government might find it beneficial to devote more efforts towards promoting maternity services, especially targeting women of lower economic status at fertile ages. The government needs to develop and implement a reasoned strategy to ensure stable and long-term funding of health services and to increase their availability to all socio-economic layers of society.

However, there are some inherent limitations to our study design that should be considered when interpreting the results. First, all the data were collected by a self-report approach, and there may be recall-bias. However, in both surveys, the data was collected within one year of the childbirth under investigation. Pregnancy and childbirth are events that women remember for years, thus the recall bias is assumed to be small. Second, we relied solely on the numerical value of family income to indicate the family’s economic index. This feature may have been insufficient to accurately measure a family’s wealth. For example, a family may have had other sources of wealth, such as property, that would not be reflected by yearly income. Thus, during the portion of the interview (using the structured questionnaire) that was related to income and expenditures, the interviewers asked: “compared with other families in your village, how is your economic status rated on a 5-degree scale?” We then verified the economic levels by the reported income and expenditures, respectively, and found that the relative levels were similar. Third, the time for giving a survey must be considered in order to lessen the rate of non-response. In 2007, the survey was conducted after the national Spring Festival, and the higher non-responder rate (vs. in 2008 – 2009) was reported by the local doctors to be due to the general practice of travel during this time. Then in 2008–2009, the part of the survey that was conducted before the Spring Festival had completed. In general, the incomes of families with an employed mother is better than families with a mother who is unemployed, and an inaccurate response rate for this parameter may impact the study’s results (i.e. underestimating the inequity). Finally, the maternal mortality rate is generally considered a good index of a women’s health. Unfortunately, the sample sizes of our surveys were too small to analyze that particular index and larger study populations are needed. In the current study, the CI of combined data from both counties was similar to the CIs that were calculated for each county in isolation. For example, the inequity in deliveries occurring at a healthcare facility showed the same statistical significant in each year and in each county. Similar results were also obtained for the analyses of antenatal care within 12 weeks and proportion of home deliveries. Certainly, future equity analyses need to be nuanced enough to identify the remaining disparities between different types of rural and urban areas, in addition to the broad rural–urban inequities.

## Conclusions

It can be concluded that inequity in the use of maternity care still exists in the rural Shaanxi province of China. The index of inequity in both antenatal care and postnatal visits seemed more serious in low-income families. Therefore, economic level is still one of the main causes of inequity for utilization of maternal health services. Increasing the accessibility and availability of health care services are important for improving the general maternal health in populations of rural areas in China. Financial support that enables the poorer population of pregnant women to use health services will be beneficial, as utilization of maternal healthcare services may be improved if out-of-pocket expenses can be minimized. At the same time, effective demand generation strategies are also necessary to encourage women to utilize health facilities. It is important for the government to consider how to make the best use of limited funds to maximize the level of maternal and child health care.

## Abbreviations

CI: Concentration index; MCH: Maternal and child health; MMR: Maternal mortality rate; NCMS: New cooperative medical scheme; PPS: Probability proportionate to size.

## Competing interests

The authors declare that they have no competing interests.

## Authors’ contributions

YS prepared the research protocol, performed field management and coordination, collected and analyzed the data, and wrote the manuscript. HY designed the research study, and consulted on the data analysis. KR arranged the study’s implementation, providing instructions on subject-related interactions, and contributed to preparation of the manuscript. QL modified the manuscript for important intellectual content and grammatical clarity. SX performed field management and contributed to preparation of the research protocol as well as collection and analysis of the data. JG contributed to preparation of the research protocol, performed field management, and contributed to implementation of the equity method and index calculations as well as interpretation of the results. ZZ contributed to the writing of the manuscript and provided guidance in the analysis. All authors read and approved the final manuscript.

## Pre-publication history

The pre-publication history for this paper can be accessed here:

http://www.biomedcentral.com/1472-6963/14/155/prepub
